# A EWTD Compliant Rotation Schedule Which Protects Elective Training Opportunities Is Safe and Provides Sufficient Exposure to Emergency General Surgery: A Prospective Study

**DOI:** 10.1155/2015/735129

**Published:** 2015-11-02

**Authors:** Andrew Emmanuel, Ezzat Chohda, Carolyn Sands, Joseph Ellul, Hamid Khawaja

**Affiliations:** Department of General Surgery, Princess Royal University Hospital, Kings College NHS Foundation Trust, Farnborough Common, Orpington, Kent BR6 8ND, UK

## Abstract

*Introduction*. Training opportunities have decreased dramatically since the introduction of the European Working Time Directive (EWTD). In order to maximise training we introduced a rotation schedule in which registrars do not work night shifts and elective training opportunities are protected. We aimed to determine the safety and effectiveness of this EWTD compliant rotation schedule in achieving exposure of trainees to acute general surgical admissions and operations. *Methods*. A prospective study of consecutive emergency surgical admissions over a 6-month period. Exposure to acute admissions and operative procedures and patient outcomes during day and night shifts was compared. *Results*. There were 1156 emergency admissions covering a broad range of acute conditions. Significantly more patients were admitted during the day shift and almost all emergency procedures were performed during the day shift (2.1 versus 0.3, *p* < 0.001). A registrar was the primary operating surgeon in 49% of cases and was directly involved in over 65%. There were no significant differences between patients admitted during the day and night shifts in mortality rate, length of stay, admission to ICU, requirement for surgery, or readmission rates. *Conclusion*. A EWTD compliant rotation schedule that protects elective training opportunities is safe for patients and provides adequate exposure to training opportunities in emergency surgery.

## 1. Introduction

There continues to be heated debate around the effects of the European Working Time Directive (EWTD) on surgical training. The Royal College of Surgeons of England (RCSEng) and surgical trainee organisations strongly advocate opting out of the EWTD and extending working hours [1–3]. Although some studies have not found significant reductions in exposure to operative procedures after the introduction of working time restrictions [4], the majority of published studies conclude that training opportunities and operative exposure for trainees have decreased dramatically since the introduction of the EWTD [5–14]. However, many of these studies are based only on trainee questionnaires and surveys or retrospective logbook reviews. As a result, most studies tend to be based on the views of trainees rather than actual data on trainees' exposure. Furthermore, most studies do not take into account many other factors that have had an important impact on operative training volume such as increases in the number of trainees and changes in surgical management and practice. They also tend to concentrate only on operative experience, which is merely one of several skills required of a surgeon. Exposure to other training opportunities such as assessment of acute surgical patients is generally not assessed by these studies. Furthermore, most studies have not compared this perceived reduced exposure to any standard indicative number of procedures that should be achieved.

The RCSEng has previously recommended that, in order to maximise training opportunities, senior trainees should not work full shifts at night unless a significant opportunity for training exists [15]. Night shifts in surgical specialities such as trauma and orthopaedics clearly provide minimal opportunities for training, but it is less clear what opportunities exist during the night shift in general surgery which has acutely unwell patients, some requiring urgent surgery, presenting unpredictably at any time. As a result of the RCSEng recommendations, our institution introduced a EWTD compliant rotation schedule in which senior trainees do not work full night shifts and therefore do not require compensatory rest periods and are available to attend their normal elective theatre and endoscopy lists and clinics. As no elective opportunities are missed with this system, the only potential problem is lack of exposure to emergency general surgery.

The aim of our study was to determine the safety and effectiveness of a EWTD compliant rotation schedule, which retains full exposure to elective opportunities, in achieving exposure of trainees to acute general surgical admissions and operations.

## 2. Methods

Our institution has adopted an emergency rotation schedule for registrars which is EWTD compliant but avoids a full shift pattern in order to protect day-time exposure to training opportunities. Registrars work an emergency day-time shift from 08:00 to 21:00. The night shift (21:00–08:00) is covered by two senior house officers with a senior nontraining middle grade surgeon and consultant surgeon on call but off-site.

A prospective study was undertaken of all consecutive emergency general surgical admissions over a 6-month period from 27 January 2012 to 26 July 2012. Data on significant events during the hospital stay such as the need for surgery or admission to the intensive care unit (ICU) was obtained from patients' records and data on the timing of emergency procedures and operating surgeon were obtained from electronic and written theatre logs. Data collected included time of admission, diagnosis, length of stay (LOS), admission to ICU, readmission within 30 days of discharge, in-hospital mortality, emergency procedures and the time they were performed, the grade of the primary surgeon performing the procedures, and the grade of assistant. Admissions, outcomes, and operative procedures were compared between day shifts (08:00–21:00) and night shifts (21:00–08:00). Means were compared using one-way analysis of variance (ANOVA) or Mann-Whitney *U* test for nonnormally distributed data and proportions compared using the chi-squared test.

## 3. Results

There were 1156 emergency general surgery admissions over the study period. The mean age of the patients was 55 years and 58% were female. The diagnoses on admission covered a broad range of emergency general surgical conditions (Table 1). The majority of patients were admitted during the day shift with few admitted during the night shift (mean 4.9 versus 1.6, *p* < 0.001).

Almost all patients requiring an emergency procedure had this performed during the day shift with very few operations carried out during the night shift (mean 2.1 versus 0.3 procedures, *p* < 0.001).  Table 2 shows there were a broad range of emergency procedures performed which included most emergency procedures to which general surgical trainees would be expected to gain exposure. Very few operative training opportunities were lost by not working night shifts.

There were no significant differences in a variety of outcomes between patients admitted during the day and night shifts, including mortality rate, length of hospital stay, 30-day readmission rate, the need for intensive care admission, or the need for an emergency surgical procedure (Table 3).

For the emergency procedures performed during the day shift, a registrar was the primary operating surgeon in 49% of cases and there was a consultant or associate specialist assisting the registrar in 24% of these cases. A registrar was directly involved in over 65% of cases.

## 4. Discussion

Although there has been debate about the effect of the EWTD on surgical training, the bulk of published opinion is that the EWTD has led to substantial decreases in the quantity and quality of surgical training. These studies largely cite significantly reduced numbers of procedures being performed by trainees following the introduction of the EWTD compared to traditional work patterns and conclude therefore that there is a deficit in training.

However, there are a number of problems with many of the published studies to date and the reasoning that has led to a broad acceptance of the idea that surgical training in the EWTD era is inadequate. For example, many studies that show reduced operative experience for trainees are based on retrospective analysis of surgeons logbooks and operative logs or questionnaires applied to trainees and conclusions about training extrapolated from these [5, 14]. Logbooks do not give a full picture of training and clearly questionnaires merely gauge the prevailing opinion amongst trainees without being based on reliable data. Also, these studies do not take into account the totality of emergency surgery training which in large part involves the assessment of acute surgical patients in the emergency department, making diagnostic decisions and initiating appropriate management. There are few if any prospective studies which assess exposure to the emergency general surgical take as a whole, including exposure to the range of acute general surgical conditions as well as to emergency procedures, with a EWTD compliant rotation schedule.

Another problem with the conclusions drawn from many of the studies critical of the EWTD is that they can be seen as overly simplistic. A finding of reduced operative numbers amongst trainees has many potential explanations other than simply poor training in a EWTD era. There is no doubt that surgical practice is changing. The type and number of operative procedures are changing, with trends in many areas toward conservative management of conditions which in the past were thought to mandate surgery and minimally invasive techniques in other areas. For example, one US study showed a trend toward major increases in percutaneous techniques and sharp declines in traditional open surgical techniques [16]. One result of the increased use of minimally invasive techniques is that consultant surgeons are likely to perform more procedures that would traditionally have been carried out by a trainee when doing an open operation [17]. The duties and competencies expected of a modern surgeon have also changed. Surgeons qualifying in the current era take on a more subspecialised role than surgeons in the past who may have required greater operative volume of a broader range of procedures to fulfil their role. There is also a trend toward greater consultant lead and delivered care with less reliance on trainees to deliver patient care than in the past [18]. Rather than all effects on procedure volume for trainees being attributable to EWTD changes, these factors may also impact on the numbers of procedures being performed by trainees but could have significant positive effects on the quality of training and the skill set that modern surgeons require. For example, very few studies concluding that the EWTD has had a negative impact on training consider changes to the levels of consultant supervision of trainees performing procedures. There is evidence that consultant supervision of registrars performing procedures has increased dramatically after the introduction of the EWTD [19]. This will clearly have an impact on the quality of training and it is feasible that significantly fewer procedures are required to gain competence if there is quality training with adequate supervision, an issue which most studies do not address.

The Royal College of Surgeons of England recommends that, in order to protect training in the EWTD era, full shift working should be avoided wherever possible for senior trainees (ST3 and above) and that senior trainees should only work as part of a full shift system if it is required for training purposes [15]. This view is supported by other professional surgical bodies [20]. It therefore seems reasonable that for craft specialties such as surgery there is little to be gained from a training perspective by working night shifts. However, whilst this may more definitively be the case for disciplines such as orthopaedic surgery, a general surgical emergency take involves the assessment and management of acute patients who can present at any time of day or night and potentially require emergency surgery as well. Hence, it is necessary to evaluate the level of exposure and therefore potential training, to the broad range of emergency general surgical conditions and procedures during day and night shifts.

Our institution has been using a rotation schedule that is EWTD compliant but ensures that trainees do not work night shifts and are therefore not subject to the mandatory periods of rest which follow night shifts. As a result, they are available to attend all of the firm's elective commitments such as elective surgery, endoscopy, and clinics. Elective training opportunities are therefore unaffected by this rotation schedule, and the only issue is whether there is adequate exposure to emergency general surgical patients and procedures and furthermore whether such a system represents safe practice. Following the NCEPOD report [17] and in common with many other UK hospitals, our institution runs a separate, dedicated emergency theatre which is staffed by dedicated on call anaesthesiology, theatre nurse, and on call general surgery teams. This allows cases requiring surgery presenting at night that are not limb- or life-threatening to be deferred until the day shift while eliminating the struggle to find theatre time and staff to accommodate such cases on a daily basis.

Our study shows that this rotation schedule is safe with no difference between patients admitted during the night shift and those admitted during the day shift in in-hospital mortality, admission to ICU, length of hospital stay, or 30-day readmission rates. It shows that patients with a broad range of emergency general surgical conditions present during the day shift and, in comparison, very few present during the night shift. Similarly, the vast majority of operative exposure is to be gained during the day shift with very few operations performed during the night shift. Very few training opportunities are missed as a result of not working a night shift. In addition, if the exposure to emergency procedures in this study is indicative of the type of exposure a trainee can expect during the course of their training, this rotation schedule should provide sufficient exposure to emergency surgery.

Although several surgical bodies advocate against the EWTD, it is likely to remain in force for the foreseeable future and most trainees will complete their training under this system. It is therefore imperative that innovative solutions are sought to protect training. Such solutions include improved training and supervision with a focus on quality rather than quantity [21], the use of surgical simulation [22, 23], improvements in surgical curricula [24], and, perhaps most importantly, innovations in emergency rotation schedules for trainees to protect exposure to elective training opportunities whilst allowing adequate training in the emergency take and procedures in comparison to trainees at the end of their training applying for a certificate of completion of training [25, 26]. Our study supports the notion that innovative rotation schedules can protect all training opportunities [27].

We recognise some limitations of our study. It is difficult to accurately predict the mortality and morbidity risk of this patient population who presented with a variety of acute surgical conditions and underwent different management strategies. Many were treated nonoperatively. As a result, we did not perform any risk adjustment calculations for patients admitted during the day and night shifts. However, we would not expect this to significantly affect our conclusions as intuitively we would expect patients presenting at night to be more acutely unwell than those presenting during the day, but we did not find that these patients experienced worse outcomes using our rotation schedule. Another limitation is the paucity of trauma cases at our institution. However, trauma cases in the UK are now transferred directly to a limited number of nominated major trauma centres and so our experience is typical of an acute district hospital general surgery service. Departments such as ours provide the bulk of emergency general surgery care in the UK and general surgery trainees who do not elect to have an interest in major trauma would spend most of their training time in a similar setting.

We conclude that a EWTD compliant rotation schedule that protects elective opportunities is safe for patients and provides adequate exposure to training opportunities in emergency surgery.

## Figures and Tables

**Table 1:** Emergency general surgical admissions.

Diagnosis	Frequency
Biliary disease	137
Appendicitis	103
Abscess/soft tissue infection	98
GI bleed	145
Bowel obstruction	73
Pancreatitis	45
Diverticulitis	44
Constipation/pseudoobstruction	39
Hernias	37
Abdominal pain	238
Trauma	19
Malignancy	27
Perforated viscus	13
Intra-abdominal sepsis	10
Postoperative complication	63
IBD	16
Other surgical diagnosis	29
Gynaecology problem	9
Medical problem	11
Total	1156

**Table 2:** Emergency procedures performed overall and during the day shift.

Procedure	Total	Day shift
Appendicectomy	120	101
Endoscopy (CEPOD)	117	98
I&D	76	66
Laparotomy	63	51
Hernia repair	26	23
Laparoscopy	23	22
Hartmann's procedure	9	6
Small bowel resection	22	19
Large bowel resection	10	9
Adhesiolysis	15	10
Stoma	9	8
Cholecystectomy	8	8
Other	25	23

**Table 3:** Comparison of outcomes between patients admitted during day and night shifts.

	Shift admitted		*p* value
	Day	Night	
Mortality (%)	3.5	4.3	0.57
ICU admission (%)	2.2	3.2	0.32
Mean length of stay (days)	5.1	5.7	0.18^*∗*^
Readmission, 30 days (%)	8.1	7.1	0.60
Need for surgery (%)	43.5	39.6	0.26

^*∗*^Mann-Whitney *U* test.
